# An efficient linear mixed model framework for meta-analytic association studies across multiple contexts

**DOI:** 10.4230/LIPIcs.WABI.2021.10

**Published:** 2021

**Authors:** Brandon Jew, Jiajin Li, Sriram Sankararaman, Jae Hoon Sul

**Affiliations:** Bioinformatics Interdepartmental Program, University of California, Los Angeles, USA; Department of Human Genetics, University of California, Los Angeles, USA; Department of Human Genetics, University of California, Los Angeles, USA; Department of Computer Science, University of California, Los Angeles, USA; Department of Computational Medicine, University of California, Los Angeles, USA; Department of Psychiatry and Biobehavioral Sciences, University of California, Los Angeles, USA

**Keywords:** Meta-analysis, Linear mixed models, multiple-context genetic association, Applied computing → Bioinformatics, Applied computing → Computational genomics

## Abstract

Linear mixed models (LMMs) can be applied in the meta-analyses of responses from individuals across multiple contexts, increasing power to detect associations while accounting for confounding effects arising from within-individual variation. However, traditional approaches to fitting these models can be computationally intractable. Here, we describe an efficient and exact method for fitting a multiple-context linear mixed model. Whereas existing exact methods may be cubic in their time complexity with respect to the number of individuals, our approach for multiple-context LMMs (mcLMM) is linear. These improvements allow for large-scale analyses requiring computing time and memory magnitudes of order less than existing methods. As examples, we apply our approach to identify expression quantitative trait loci from large-scale gene expression data measured across multiple tissues as well as joint analyses of multiple phenotypes in genome-wide association studies at biobank scale.

## 1 Introduction

Over the last decade, the scale of genomic datasets has steadily increased. These datasets have grown to the size of hundreds of thousands of individuals [3] with millions soon to come [21]. Similarly, datasets for transcriptomics and epigenomics are growing to thousands of samples [1, 5, 14]. These studies provide valuable insight into the relationship between our genome and complex phenotypes [23].

Identifying these associations requires statistical models that can account for biases in study design that can negatively influence results through false positives or decreased power. Linear mixed models (LMMs) have been a popular choice for controlling these biases in genomic studies, utilizing variance components to account for issues such as population stratification [8]. These models can also be used to analyze studies with repeated measurements from individuals, such as replicates or measurements across different contexts. Meta-Tissue [20] is a method that applies this model in the context of identifying expression quantitative trait loci (eQTLs) across multiple tissues. In this framework, gene expression is measured in several tissues from the same individuals and the LMM is utilized to test the association between these values and genotypes. A meta-analytic approach is used to combined effects across multiple tissues to increase the power of detecting eQTLs. This approach has also been applied to increase power in genome-wide association studies (GWAS) by testing the association between genotypes and multiple related phenotypes [7].

However, these approaches are computationally intensive. Existing approaches for fitting these models are cubic in time complexity with respect to the number of samples across all contexts [8, 26]. Here, we present an ultra-fast LMM framework specifically for multiple-context studies. Our method, mcLMM, is linear in complexity with respect to the number of individuals and allows for statistical tests in a manner of hours rather than days or years with existing approaches. To illustrate the computational efficiency of mcLMM, we compare the runtime and memory usage of our method with EMMA and GEMMA [8, 26], two popular approaches for fitting these models. We further apply mcLMM to identify a large number of eQTLs in the Genotype-Tissue Expression (GTEx) dataset [5] and compare our results from METASOFT [6], which performs the meta-analysis of the mcLMM output, to a recent meta-analytic approach known as mash [22]. Finally, to demonstrate the practicality of mcLMM on modern datasets, we perform a multiple-phenotype GWAS combining over a million observations sampled from hundreds of thousands of individuals in the UK Biobank [3] within hours.

## 2 Methods

### 2.1 Linear Mixed Model

For multiple-context experiments with *n* individuals, *t* contexts, and *c* covariates, we fit the following linear mixed model

(1) y=Xβ+u+e

Where $u∼N\left(0,\sigma_{g}^{2}K\right),e∼N\left(0,\sigma_{e}^{2}I\right),y\in R^{nt}$ is a vectorized representation of the responses, $X\in R^{nt\times tc}$ is the matrix of covariates, $\beta \in R^{tc}$ is the vector of estimated coefficients $K\in R^{nt\times nt}$ is a binary matrix where $K_{i,j}=1$ indicates that sample *i* and sample *j* in *Y* come from the same individual, and $I\in R^{nt\times nt}$ is an identity matrix. *X* is structured such that both an intercept and the covariate effects are fit within each context. For sake of simplicity, dimensions of *nt* assume that there is no missing data; however, this is not a requirement for the model. We note that this definition of *K* models within-individual variability as a random-effect, while within-context or across-individual variability is not included.

The full and restricted log-likelihood functions for this model are

(2) $$l_{F}\left(y;\beta ,\sigma_{g},\delta \right)=\frac{1}{2}\left[-N\log\left(2\pi \sigma_{g}^{2}\right)-\log(|H|)-\frac{1}{\sigma_{g}^{2}}{\left(y-X\beta \right)}^{T}H^{-1}(y-X\beta )\right]$$

(3) $$l_{R}\left(y;\beta ,\sigma_{g},\delta \right)=l_{F}\left(y;\beta ,\sigma_{g},\sigma_{e}\right)+\frac{1}{2}\left[tc\log\left(2\pi \sigma_{g}^{2}\right)+\log\left(\left|X^{T}X\right|\right)-\log\left(\left|X^{T}H^{-1}X\right|\right)\right]$$

where *N* is the total number of measurements made across the individuals and contexts $\delta =\frac{\sigma_{e}^{2}}{\sigma_{g}^{2}}$ and H=K+δI [24]. These likelihood functions are maximized with the generalized least squares estimator $\hat{\beta }={\left(X^{T}H^{-1}X\right)}^{-1}X^{T}H^{-1}y$ and ${\hat{\sigma }}_{g}^{2}=\frac{R}{N}$ in the full log-likelihood and ${\hat{\sigma }}_{g}^{2}=\frac{R}{N-tc}$ in the restricted log-likelihood, where $R={\left(y-X\hat{\beta }\right)}^{T}H^{-1}(y-X\hat{\beta })$ Our goal is to maximize these likelihood functions to estimate the optimal $\hat{\delta }$.

### 2.2 Likelihood refactoring in the general case

The EMMA algorithm optimizes these likelihoods for *δ* by refactoring them in terms of constants calculated from eigendecompositions of *H* and *SHS*, where $S=I-X{\left(X^{T}X\right)}^{-1}X^{T}$, that allow linear complexity optimization iterations with respect to the number of individuals [8]. The GEMMA algorithm further increases efficiency by replacing the *SHS* eigendecomposition with a matrix-vector multiplication [26]. Both approaches require the eigendecomposition of at least one *N* by *N* matrix which is typically cubic in complexity. Here, we show that our specific definition of *K* as a binary indicator matrix allows us to refactor these likelihood functions without any eigendecomposition steps. It should be noted that EMMA and GEMMA can fit this model for any positive semidefinite K, while mcLMM is restricted to the definition described above.

We note that previous work has shown similar speedups when the matrix *K* is low rank and has a block structure as described here [10]. This work, FaST-LMM, shows that the likelihood functions can be computed in linear time with respect to the number of individuals after singular value decomposition of a matrix with complexity that is also linear with respect to the number of individuals. We improve upon these methods by recognizing that the eigenvalues of the *K* matrix are known beforehand, which allows for further efficiency in fitting this model. Furthermore, the FaST-LMM model assumes that all individuals within each context share additional covariance while mcLMM assumes that all contexts observed within an individual share additional covariance.

First, note that *H = K+δI* is a block diagonal matrix. Specifically, each block corresponds to an individual *i* with *t*_*i*_ contexts measured, where *t*_*i*_ is less than or equal to *t* depending on the number of contexts observed for individual *i*. Each block is equal to $\left[1_{t_{i}}+\delta I_{t_{i}}\right]\in R^{t_{i}\times t_{i}}$ where 1_*ti*_ is a *t*_*i*_ by *t*_*i*_ matrix composed entirely of 1. These properties of *H* make its eigendecomposition and inverse directly known.

The eigenvalues of a block diagonal matrix are equal to the union of the eigenvalues of each block. Moreover, the eigenvalues of $\left[1_{t_{i}}+\delta I_{t_{i}}\right]$ are $t_{i}+\delta$ with multiplicity 1 and *δ* with multiplicity *t*_*i*_ - 1. Therefore, *H* has eigenvalues *δ* with multiplicity *N* - *n* and *t*_*i*_ + *δ* for each *t*_*i*_. This provides our first refactoring

(4) $$\log(|H|)=(N-n)\log(\delta )+\sum_{i=1}^{n}\log\left(t_{i}+\delta \right)$$

The inverse of a block diagonal matrix can also be computed by inverting each block individually. Moreover, using the Sherman-Morrison formula [16], the inverse of [1_*t_*i*_*_ + *δI*_*t_*i*_*_] is known

(5) $${\left(1_{t_{i}}+\delta I_{t_{i}}\right)}^{-1}=-\frac{1}{t+\delta }1_{t_{i}}+\frac{1}{\delta }I_{t_{i}}$$

Given each entry of *H*^−1^, we can show algebraically that

(6) $$X^{T}H^{-1}X=\frac{1}{\delta }(E-D)$$

(7) $$E_{i,j}=\{\begin{array}{cc} \sum_{\text{ind}\in f(i)}x_{\text{ind},g(i)}x_{\text{ind},g(j)} & \text{if }f(i)=f(j)\\ 0 & \text{if }f(i)\neq f(j) \end{array}$$

(8) $$D_{i,j}=\sum_{g\in \text{ groups }}\frac{1}{t_{g}+\delta }\sum_{\text{ind}\in f(i),f(j),g}x_{\text{ind},g(i)}x_{\text{ind},g(j)}$$

where *f* (*i*) = *i%t* (modulo operator) provides the context of a given 0-indexed column of X, *g*(*i*) = *i//t* (integer division) provides the covariate of a given index. A group *g* defines the set of individuals that share the same number of measured contexts *t*_g_. The expression “ind $\in f(i),f(j),g^{\prime \prime }$ indicates the set of all individuals that have *t*_*g*_ measured contexts that include context *i* and *j*.

Note that with all values independent of *δ* pre-computed, specifically the sum of covariate interactions within the sets of individuals indicated above, *E* is constant with respect to *δ* and each entry of the symmetric matrix *D* can be calculated in linear time with respect to the number of groups, which is less than or equal to the number of contexts *t*. For a given δ, we can compute *X*^*T*^*H*^*−1*^*X* in *O*(*t*(*tc*)^2^) time complexity. Both the restricted and full log-likelihoods require the calculation of (*X*^*T*^*H*^−1^*X*)^−1^. The restricted log-likelihood requires the additional calculation of $\log\left(\left|X^{T}H^{-1}X\right|\right)$. To calculate both of these terms, we perform a Cholesky decomposition of $X^{T}H^{-1}X=LL^{*}$, where * indicates the conjugate transpose. Given this decomposition, we can compute

(9) $$\log\left(\left|X^{T}H^{-1}X\right|\right)=\sum_{i=1}^{tc}2\log\left(L_{i,i}\right)$$

(10) $${\left(X^{T}H^{-1}X\right)}^{-1}={\left(L^{*}\right)}^{-1}L^{-1}$$

These operations can be done in *O*((*tc*)^*3*^) time complexity.

Let *P*(*X*) denote a projection matrix and *M*(*X*) *=* (*I — P*(*X*)). Note that both *P*(*X)* and *M* (*X*) are idempotent. The term remaining term in the likelihood functions, *R,* can be reformulated as follows

(11) $$y-X\hat{\beta }=y-X{\left(X^{T}H^{-1}X\right)}^{-1}X^{T}H^{-1}y\;=\left(I-X{\left(X^{T}H^{-1}X\right)}^{-1}X^{T}H^{-1}\right)y\;=(I-P(X))y\;=M(X)y$$

(12) $$M{\left(X\right)}^{T}H^{-1}={\left(I-X{\left(X^{T}H^{-1}X\right)}^{-1}X^{T}H^{-1}\right)}^{T}H^{-1}\;=\left(I-H^{-1}X{\left(X^{T}H^{-1}X\right)}^{-1}X^{T}\right)H^{-1}\;=H^{-1}-H^{-1}X{\left(X^{T}H^{-1}X\right)}^{-1}X^{T}H^{-1}\;=H^{-1}\left(I-X{\left(X^{T}H^{-1}X\right)}^{-1}X^{T}H^{-1}\right)\;=H^{-1}M(X)$$

(13) $$R={\left(y-X\hat{\beta }\right)}^{T}H^{-1}(y-X\hat{\beta })\;=y^{T}M{\left(X\right)}^{T}H^{-1}M(X)y\;=y^{T}H^{-1}M(X)M(X)y\;=y^{T}H^{-1}M(X)y\;=\left(y^{T}H^{-1}y\right)-\left(y^{T}H^{-1}X{\left(X^{T}H^{-1}X\right)}^{-1}X^{T}H^{-1}y\right)\;=a-b^{T}{\left(X^{T}H^{-1}X\right)}^{-1}b\;=a-b^{T}{\left(L^{*}\right)}^{-1}L^{-1}b$$

The scalar *a* and vector b are a function of *δ* and can be algebraically formulated as

(14) $$a=\frac{1}{\delta }\left(\left(\sum_{i=1}^{N}y_{i}^{2}\right)-\left(\sum_{g\in \text{ groups }}\frac{1}{t_{g}+\delta }\sum_{\text{ind}\in g}{\left(\sum y_{\text{ind}}\right)}^{2}\right)\right)$$

(15) $$b_{i}=\frac{1}{\delta }\left(\left(\sum_{\text{ind}\in \text{ context }(\text{i})}x_{\text{ind},g(i)}y_{\text{ind},f(i)}\right)-\left(\sum_{g\in \text{ groups }}\frac{1}{t_{g}+\delta }\sum_{\text{ind}\in f(i),g}x_{\text{ind},g(i)}\left(\sum y_{\text{ind}}\right)\right)\right)$$

where $\sum y_{\text{ind}}$ indicates the sum of responses across all contexts for an individual. With values independent of *δ* pre-calculated, *a* and b can be calculated in linear time with respect to the number of groups.

Note that Equations 16 and 17 remove terms that are independent of *δ* since they are not required for finding its optimal value, indicated by the ≈ symbol. We can reformulate the entire likelihood functions as follows

(16) $$l_{F}\left(y;\beta ,\sigma_{g},\delta \right)=\frac{1}{2}\left[-N\log\left(2\pi \sigma_{g}^{2}\right)-\log(|H|)-\frac{1}{\sigma_{g}^{2}}{\left(y-X\beta \right)}^{T}H^{-1}(y-X\beta )\right]\;=\frac{1}{2}\left[-N\log\left(2\pi \frac{R}{N}\right)-\log(|H|)-N\right]\;=\frac{1}{2}\left[-N\log\left(2\pi \frac{R}{N}\right)-\left((N-n)\log(\delta )+\sum_{i=1}^{n}\log\left(t_{i}+\delta \right)\right)-N\right]\;\approx -N\log\left(a-b^{T}{\left(L^{*}\right)}^{-1}L^{-1}b\right)-\left((N-n)\log(\delta )+\sum_{i=1}^{n}\log\left(t_{i}+\delta \right)\right)$$

(17) $$l_{R}\left(y;\beta ,\sigma_{g},\delta \right)=l_{F}\left(y;\beta ,\sigma_{g},\sigma_{e}\right)+\frac{1}{2}\left[tc\log\left(2\pi \sigma_{g}^{2}\right)+\log\left(\left|X^{T}X\right|\right)-\log\left(\left|X^{T}H^{-1}X\right|\right)\right]\;\approx (tc-N)\log\left(a-b^{T}{\left(L^{*}\right)}^{-1}L^{-1}b\right)\;-\left((N-n)\log(\delta )+\sum_{i=1}^{n}\log\left(t_{i}+\delta \right)\right)-\sum_{i=1}^{tc}2\log\left(L_{i,i}\right)$$

These likelihoods are maximized for $\hat{\delta }$ using the optimize function in R. Each likelihood evaluation has a time complexity of *O*((*tc*)^3^ + *n*).

### 2.3 Likelihood refactoring with no missing data

When there is no missing data, the likelihood functions can be further simplified. Note that in this case, *N* = *nt* and all *t*_*i*_ = *t*. Hence,

(18) $$\log(|H|)=(N-n)\log(\delta )+\sum_{i=1}^{n}\log\left(t_{i}+\delta \right)\;=(nt-n)log(\delta )+n\log(t+\delta )$$

If the input terms y, *X*, and *K* are permuted resulting in samples being sorted in order of context, and the covariates in *X* are sorted in order of context, we can decompose *H* and *X* into

(19) $$H=\left(1_{t}+\delta I_{t}\right)⊗I_{n}$$

(20) $$X=I_{t}⊗X_{\text{dense }}$$

where ⊗ indicates the Kronecker product and $X_{\text{dense }}\in R^{n\times c}$ is a typical representation of the covariates for each individual without multiple contexts (i.e. samples as rows and covariates as columns). Utilizing the properties of Kronecker products, we can perform the following reformulation

(21) $${\left(X^{T}H^{-1}X\right)}^{-1}={\left(\left(I_{t}⊗X_{\text{dense }}^{T}\right){\left(\left(1_{t}+\delta I_{t}\right)⊗I_{n}\right)}^{-1}\left(I_{t}⊗X_{\text{dense }}\right)\right)}^{-1}\;={\left({\left(1_{t}+\delta I_{t}\right)}^{-1}⊗X_{\text{dense }}^{T}X_{\text{dense }}\right)}^{-1}\;=\left(1_{t}+\delta I_{t}\right)⊗{\left(X_{\text{dense }}^{T}X_{\text{dense }}\right)}^{-1}$$

(22) $$\log\left(\left|{\left(X^{T}H^{-1}X\right)}^{-1}\right|\right)=\log\left(\left|\left(1_{t}+\delta I_{t}\right)⊗{\left(X_{\text{dense}}^{T}X_{\text{dense }}\right)}^{-1}\right|\right)\;=\log\left({\left|\left(1_{t}+\delta I_{t}\right)\right|}^{c}{\left|{\left(X_{\text{dense }}^{T}X_{\text{dense }}\right)}^{-1}\right|}^{t}\right)\;\begin{array}{l} =c\log\left(\left|\left(1_{t}+\delta I_{t}\right)\right|\right)+t\log\left(\left|{\left(X_{\text{dense }}^{T}X_{\text{dense }}\right)}^{-1}\right|\right)\\ =c\log\left(\frac{1}{(t+\delta )\delta^{t-1}}\right)+t\log\left(\left|{\left(X_{\text{dense }}^{T}X_{\text{dense }}\right)}^{-1}\right|\right)\\ =c(-\log(t+\delta )-(t-1)\log(\delta ))+t\log\left(\left|{\left(X_{\text{dense }}^{T}X_{\text{dense }}\right)}^{-1}\right|\right) \end{array}$$

Note that the remaining determinant in Equation 22 will not need to be calculated since it is independent of *δ*. Next, we show that $\hat{\beta }$ is independent of *δ*.


(23) $$\hat{\beta }={\left(X^{T}H^{-1}X\right)}^{-1}X^{T}H^{-1}y\;=\left(\left(1_{t}+\delta I_{t}\right)⊗{\left(X_{\text{dense }}^{T}X_{\text{dense }}\right)}^{-1}\right)X^{T}H^{-1}y\;\begin{array}{l} =\left(\left(1_{t}+\delta I_{t}\right)⊗{\left(X_{\text{dense }}^{T}X_{\text{dense }}\right)}^{-1}\right)\left(I_{t}⊗X_{\text{dense }}^{T}\right)\left({\left(1_{t}+\delta I_{t}\right)}^{-1}⊗I_{n}\right)y\\ \begin{array}{c} =\left(\left(1_{t}+\delta I_{t}\right)⊗{\left(X_{\text{dense }}^{T}X_{\text{dense }}\right)}^{-1}X_{\text{dense }}^{T}\right)\left({\left(1_{t}+\delta I_{t}\right)}^{-1}⊗I_{n}\right)y\\ =\left(\left(1_{t}+\delta I_{t}\right){\left(1_{t}+\delta I_{t}\right)}^{-1}⊗{\left(X_{\text{dense }}^{T}X_{\text{dense }}\right)}^{-1}X_{\text{dense }}^{T}\right)y\\ =\left(I_{t}⊗{\left(X_{\text{dense }}^{T}X_{\text{dense }}\right)}^{-1}X_{\text{dense }}^{T}\right)y \end{array} \end{array}$$

This form of $\hat{\beta }$ shows that the optimal coefficients are equivalent to fitting separate ordinary least squares (OLS) models for each context. We compute $\hat{\beta }$ by concatenating these OLS estimates. Given this term, we can also compute the residuals of this model $s=(y-X\hat{\beta })$ and reformulate *R* as follows.


(24) $$\begin{array}{c} R={\left(y-X\hat{\beta }\right)}^{T}H^{-1}(y-X\hat{\beta })\\ =s^{T}H^{-1}s \end{array}\;=\sum_{i=1}^{nt}s_{i}\sum_{j=1}^{nt}s_{j}H_{j,i}^{-1}\;=\frac{1}{\delta }\left(\sum_{i=1}^{nt}s_{i}^{2}\right)+\frac{1}{\delta (t+\delta )}\left(-\sum_{i=1}^{n}{\left(\sum s_{\text{ind}(i)}\right)}^{2}\right)$$

The term $\sum s_{\text{ind}(i)}$ denotes the sum of residuals for an individual across all contexts. Let $u=\sum_{i=1}^{nt}s_{i}^{2}$ and $v=-\sum_{i=1}^{n}{\left(\sum s_{\text{ind}(i)}\right)}^{2}$.


(25) $$R=\frac{1}{\delta }u+\frac{1}{\delta (t+\delta )}v$$

Now we can reformulate the log-likelihoods, omitting terms that do not depend on *δ*.


(26) $$l_{F}(\delta )=-nt\log(R)-\log(|H|)\;=-nt\log\left(\frac{1}{\delta }u+\frac{1}{\delta (t+\delta )}v\right)-(nt-n)\log(\delta )-n\log(t+\delta )\;=-nt\log\left(u+\frac{1}{t+\delta }v\right)+n\log\left(\frac{\delta }{t+\delta }\right)$$

(27) $$l_{R}(\delta )=(tc-nt)\log(R)-\log(|H|)-\log\left(\left|{\left(X^{T}H^{-1}X\right)}^{-1}\right|\right)\;=(tc-nt)\log\left(u+\frac{1}{t+\delta }v\right)+(c-n)\log\left(\frac{t+\delta }{\delta }\right)$$

Both functions are differentiable with respect to *δ*. Moreover, both derivatives have the same root

(28) $$\hat{\delta }=\frac{-tu-v}{u+v}$$

The scalar values *u* and *v* can be calculated by performing a separate OLS regression for each context, which can be completed in *O*(*t*(*nc*^*2*^ *+ c*^*3*^)) time for a naive OLS implementation. Compared to the methods described above, this approach requires no iterative optimization and the estimate is optimal. Our implementation has a time complexity of *O*(*c*^*3*^ *+ nc*^*2*^ *+ tcn*).

### 2.4 Resource requirement simulation comparison

We installed EMMA v1.1.2 and manually built GEMMA from its GitHub source (genetics-statistics/GEMMA.git, commit 9c5dfbc). We edited the source code of GEMMA to prevent the automatic addition of intercept term in the design matrix (commented out lines 1946 to 1954 of src/param.cpp).

Data were simulated using the mcLMM package. Sample sizes of 100, 200, 300, 400, and 500 were simulated with 50 contexts. Context sizes of 4, 8, 16, 32, and 64 were simulated with 500 samples. Data were simulated with $\sigma_{e}^{2}=0.2$ and $\sigma_{g}^{2}=0.4$ and a sampling rate of 0.5. Memory usage of each method was measured using the peakRAM R package (v 1.0.2).

### 2.5 False positive rate simulation

We simulated gene expression levels in multiple tissues for individuals where there were no eQTLs. In other words, gene expression levels were not affected by any SNPs. We considered 10,000 genes and 100 SNPs resulting in one million gene-SNP pairs. We simulated 1,000 individuals. We also examined false positive rates with 500 and 800 individuals. We generated 49 such datasets where the number of tissues varied from 2 to 50. To simulate the genotypes for each subject, we randomly generated two haplotypes (vectors consisting of 0 and 1) assuming a minor allele frequency (MAF) of 30%. To simulate gene expression levels from multiple tissues among the same individuals, we sampled gene expression from the following multivariate normal distribution:

(29) $$y∼N\left(0,\sigma_{g}^{2}K+\sigma_{e}^{2}I\right)$$

where y is an *N* × *T* vector representing the gene expression levels of *N* individuals in *T* tissues and K is an *NT* × *NT* matrix corresponding the correlation between the subjects across the tissues. *K*_*i*_,_*j*_ *=* 1 when *i* and *j* are from two tissues of the same individuals, *K*_*i*_,_*j*_ *=* 0 otherwise. Here, we let $\sigma_{g}=\sigma_{e}=0.5$. We used a custom R function (included with the mcLMM package) to simulate data from this distribution, which is based on sampling with a smaller covariance matrix for each block of measurements from an individual.

After generating the simulation datasets, we first ran mcLMM to obtain the estimated effect sizes and their standard errors, as well as the correlation matrices. The results from mcLMM were used as the input of METASOFT for meta-analysis to evaluate the significance. False positive rate was calculated as the proportion of gene-SNP pairs with p-values smaller than the significance level (*α* = 0.05).

### 2.6 True positive simulations

We developed the true positive simulation framework based on a previous study describing mash [22]. We simulated effects for 20,000 gene-SNP pairs in 44 tissues, 400 of which have non-null effects (true positives) and 19,600 of which have null effects. Let *(β*_*jr*_ denote the effects of the gene-SNP pair *j* in context/tissue *r* and *β*_*j*_ is a vector of effects across various tissues, including null effects and non-null effects. We simulated the gene expression levels for 1,000 individuals as:

(30) $$y=\beta_{jr}^{T}X+e$$

where *X* denotes the genotypes of the individuals that were simulated as described in the false positive rate simulation. $e∼N\left(0,\sigma_{g}^{2}K+\sigma_{e}^{2}I\right)$, which is similar to the simulation in the false positive rate simulation. For *β*_*j*_, we defined two types of non-null effects and simulated them in different ways:

▀ Shared, structured effects: non-null effects are shared in all tissues and the sharing is structured. The non-null effects are similar in effect sizes and directions (up-regulation or down-regulation) across all tissues, and this similarity would be stronger among some subsets of tissues. For 19,600 null effects, we set *β*_*j*_ = 0. For 400 non-null effects, we assumed that each *β*_*j*_ independently followed a multivariate normal distribution with mean 0 and variance *wU*_*k*_, where *k* is an index number randomly sample from *1, . . . ,* 8. $\omega =\left|\omega^{\prime }\right|,\omega^{\prime }∼N(0,1)$ represents a scaling factor to help capture the full range of effects. *U*_*k*_ are 44 × 44 data-driven covariance matrices learned from the GTEx dataset, which are provided in [22].

▀ Shared, unstructured effects: non-null effects are shared in all tissues but the sharing is unstructured or independent across different tissues. For 19,600 null effects, we set *β*_*j*_ = 0. For 400 non-null effects, we sampled *β*_*j*_ from a multivariate normal distribution with mean of 0 and variance of 0.01*I*, where *I* is a 44 × 44 identity matrix.

After simulating the gene expression levels y, we first ran mcLMM on the simulated datasets to acquire the estimated effect sizes and their standard errors, as well as the correlation matrices. We then applied METASOFT for meta-analysis with mcLMM outputs to evaluate the significance. For mash, we first performed simple linear regression to get the estimates of the effects and their standard errors in each tissue separately. These estimates and standard errors were used as the inputs for mash, which returned the measure of significance for each effect, the local false sign rate (lfsr). Finally, we employed the “pROC” R package [15] to calculate the receiver operating characteristic (ROC) curve and area under the ROC curve with the significance measures (p-values for mcLMM and METASOFT, lfsr for **mash**) and the correct labels of null effects and non-null effects.

### 2.7 Analysis of the GTEx dataset

The Genotype-Tissue Expression (GTEx) v8 dataset [5] was used in this study. We down-loaded the gene expression data, the summary statistics of single-tissue cis-eQTL data using a 1 MB window around each gene, and the covariates in the eQTL analysis from GTEx portal (https://gtexportal.org/home/datasets). The subject-level genotypes were acquired from dbGaP accession number phs000424.v8.p2. The GTEx v8 dataset includes 49 tissues from 838 donors. We selected 15,627 genes that were expressed in all 49 tissues. We only included SNPs with minor allele frequency (MAF) greater than 1% and missing rate lower than 5%. We applied mash and mcLMM plus METASOFT to the GTEx v8 dataset in our analysis.

Since mash requires observation of the correlation structure among non-significant tests and data-driven covariance matrices before fitting its model, we prepared its input by selecting the top SNP with the smallest p-value and 49 random SNPs (or all other SNPs if there were fewer than 49 SNPs left in a gene) in every gene from the eQTL analysis in the GTEx v8 dataset. There were 560,475 gene-SNP pairs in total. **mash** uses the estimated effect sizes and standard errors of these gene-SNP pairs to learn the correlation structure of different conditions/tissues. We used the top significant SNPs to set up the data-driven covariances. We then fit **mash** to the random set of gene-SNP pairs with the canonical and data-driven covariances. With the fitted **mash** model, we computed the posterior summaries including local false sign rate (lfsr) [18] for the selected gene-SNP pairs to estimate the significance. We defined significant gene-SNP pairs as those with lfsr < 0.05 in any tissues.

We applied mcLMM to the same set of gene-SNP pairs. We regressed out unwanted confounding factors in gene expression levels for each tissue with a linear model using covariates provided by GTEx. Covariates of each sample included top 5 genotyping principal components, PEER factors [17] (15 factors for tissues with fewer than 150 samples, 30 factors for those with 150–250 samples, 45 factors for those with 250–350 samples, and 60 factors for those with more than 350 samples), sequencing platform, and sex. We ran mcLMM with the genotypes and processed gene expression levels of all 838 individuals across 49 GTEx tissues for each gene-SNP pair. Missing values in gene expression were included in the mcLMM input. The effect sizes, standard errors, and correlation matrices estimated by mcLMM were meta-analyzed with METASOFT to evaluate the significance under both the fixed effects (FE) and random effects (RE2) models. The resulting p-values were converted to q-values [19] to control false discovery rates. A gene-SNP pair was considered significant if its false discovery rate (FDR) was smaller than 5%.

### 2.8 Analysis of the UK Biobank dataset

This work was conducted using the UK Biobank Resource under application 33127. Samples were filtered for Caucasian individuals (Data-Field 22006)). Hard imputed genotype data from the UK Biobank were LD pruned using a window size of 50, step size of 1, and correlation threshold of 0.2. SNPs were further filtered for minor allele frequency of at least 0.01 and a Hardy-Weinberg equilibrium p-value greater than 1e-7 using Plink 2 [4]. Samples were filtered for unrelated individuals with KING using a cutoff value of 0.125 [11]. Genotype data were split by chromosome and converted to bigsnpr format (v 1.4.4) for memory efficiency [12].

The following data fields were retrieved: age at recruitment (Data-Field 31), sex (Data-Field 21022), BMI (Data-Field 23104), body fat percentage (Data-Field 23099), 10 genetic principal components (Data-Field 22009), HDL Cholesterol (Data-Field 30760), LDL Direct (Data-Field 30780), Apolipoprotein A (Data-Field 30630), Apolipoprotein B (Data-Field 30640), and Triglycerides (Data-Field 30870). Continuous phenotypes were visually inspected and triglycerides were log-transformed due to skewness. Data were filtered for complete observations. All fields were scaled to unit variance and centered at 0.

HDL cholesterol, LDL cholesterol, Apolipoprotein A, Apolipoprotein B, and triglycerides were combined as response variables in the LMM and age, sex, BMI, body fat percentage, and the top 10 genetic principal components were used as additional covariates in the model. Each SNP was marginally fit with mcLMM. The coefficients output by this model for each phenotype were meta-analyzed to calculate FE p-values using METASOFT as packaged with Meta-Tissue v 0.5. The top GWAS hits for five different chromosomes (one per chromosome) were validated using the NHGRI-EBI GWAS catalog [2] and compared to studies for LDL and HDL cholesterol (GCST008035 and GCST008037).

## 3 Results

### 3.1 mcLMM is computationally efficient

To demonstrate the efficiency of mcLMM compared to existing approaches, we applied our method to simulated data of varying sample sizes and number of contexts. For these simulations, we simulated a sampling rate of 0.5, which indicates that only half of all possible individual-context pairs of observations are expected to be sampled.

We first applied our method to simulations with a fixed number of 50 contexts and varied the sample size from 100 to 500. From these experiments, we observed that mcLMM requires computational time orders of magnitude less than EMMA and GEMMA. Similarly, when we fixed the number of samples at 500 and varied the context sizes from 4 to 64, we observed dramatically reduced runtimes for mcLMM.

**Figure 1:**
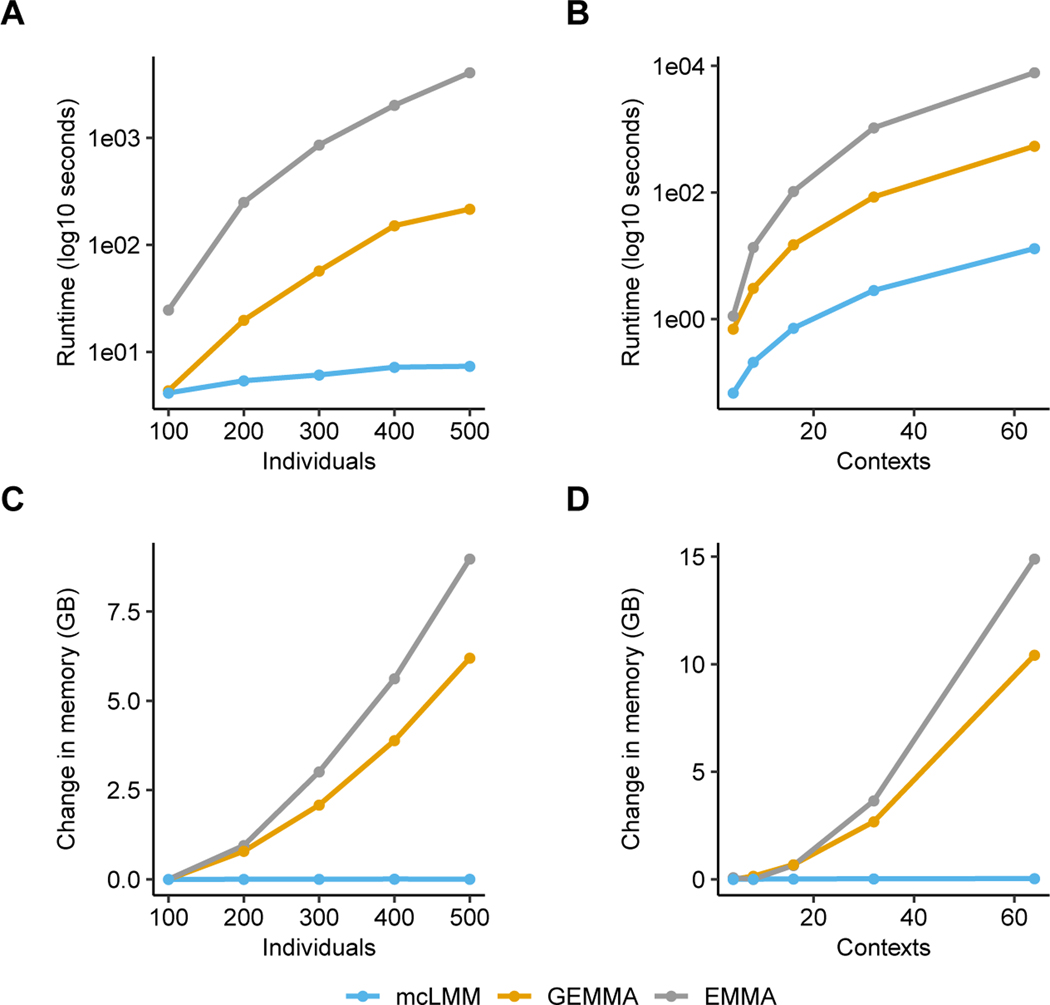
Resource requirements of mcLMM, GEMMA, and EMMA across various simulated individual and context sizes with missing values (sampling rate of 0.5). For varying individuals, contexts were fixed at 50. For varying contexts, individuals were fixed at 500. (A-B) Runtime with log10(seconds) on the y-axis and number of individuals or contexts simulated on the x-axis. (C-D) Memory usage (GB) on the y-axis and number of individuals or contexts simulated on the x-axis.

In these experiments, mcLMM also significantly reduces the memory footprint compared to EMMA and GEMMA, since we avoid creating any *nt* by *nt* matrices. In these simulations, existing approaches quickly grow memory requirements, with usages that grow to dozens of gigabytes for modestly sized datasets in the thousands of samples. mcLMM allows large-scale studies to be performed on relatively little computational resources (Figure 1).

In cases where there is no missing data, mcLMM allows for further speedups. We ran similar simulations to compare mcLMM with no missing data (optimal model) and mcLMM with missing data (iterative model). We observed a dramatic speedup, with sample sizes of 500,000 individuals across 10 contexts completed in under 10 seconds for the optimal model compared to around 15 minutes for the iterative model.

### 3.2 mcLMM enables powerful meta analyses to detect eQTLs

We utilized mcLMM to reduce the computational resource requirements of the Meta-Tissue pipeline, which fits a multiple-context LMM and combines the resulting effect sizes using METASOFT [20]. While powerful, the existing approach utilizes EMMA to fit the LMM. For a recent release from the GTEx consortium [5], each pair of genes and single nucleotide polymorphisms (SNPs) required over two hours to run. Across hundreds of thousands of gene-SNP pairs, this method would require years of computational runtime to complete. Utilizing mcLMM, we were able to complete this analysis in 3 days parallelized over each chromosome.

**Figure 2:**
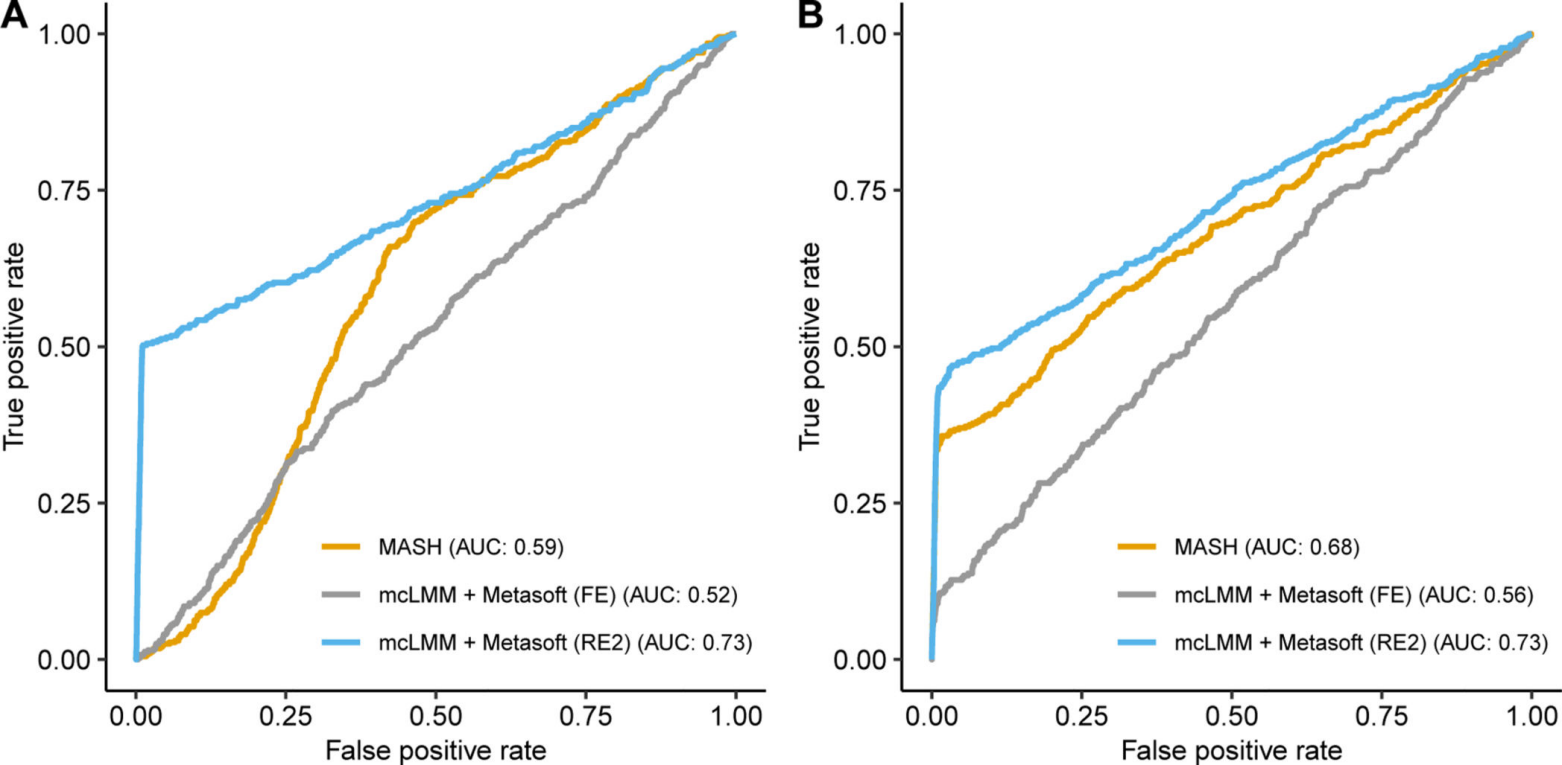
AUROC curves of mcLMM+METASOFT (fixed effects and random effects models) and **mash** in simulated data, assuming the effects of gene-SNP pairs are (A) shared and unstructured, and (B) shared and structured.

We compared our approach to a method known as mash [22]. This approach utilizes effect sizes estimated within each context independently and employs a Bayesian approach to combine their results for meta-analysis. In order to estimate the power of these methods, we performed simulations as described in the methods. In null simulations, we observed well-controlled false positive rates at *α =* 0.05 for mcLMM coupled with METASOFT. In our simulation with true positives, we observed an increased area under the receiver operating characteristic (AUROC) for mcLMM coupled with the random effects (RE2) METASOFT model compared to **mash** (Figure 2).

**Figure 3:**
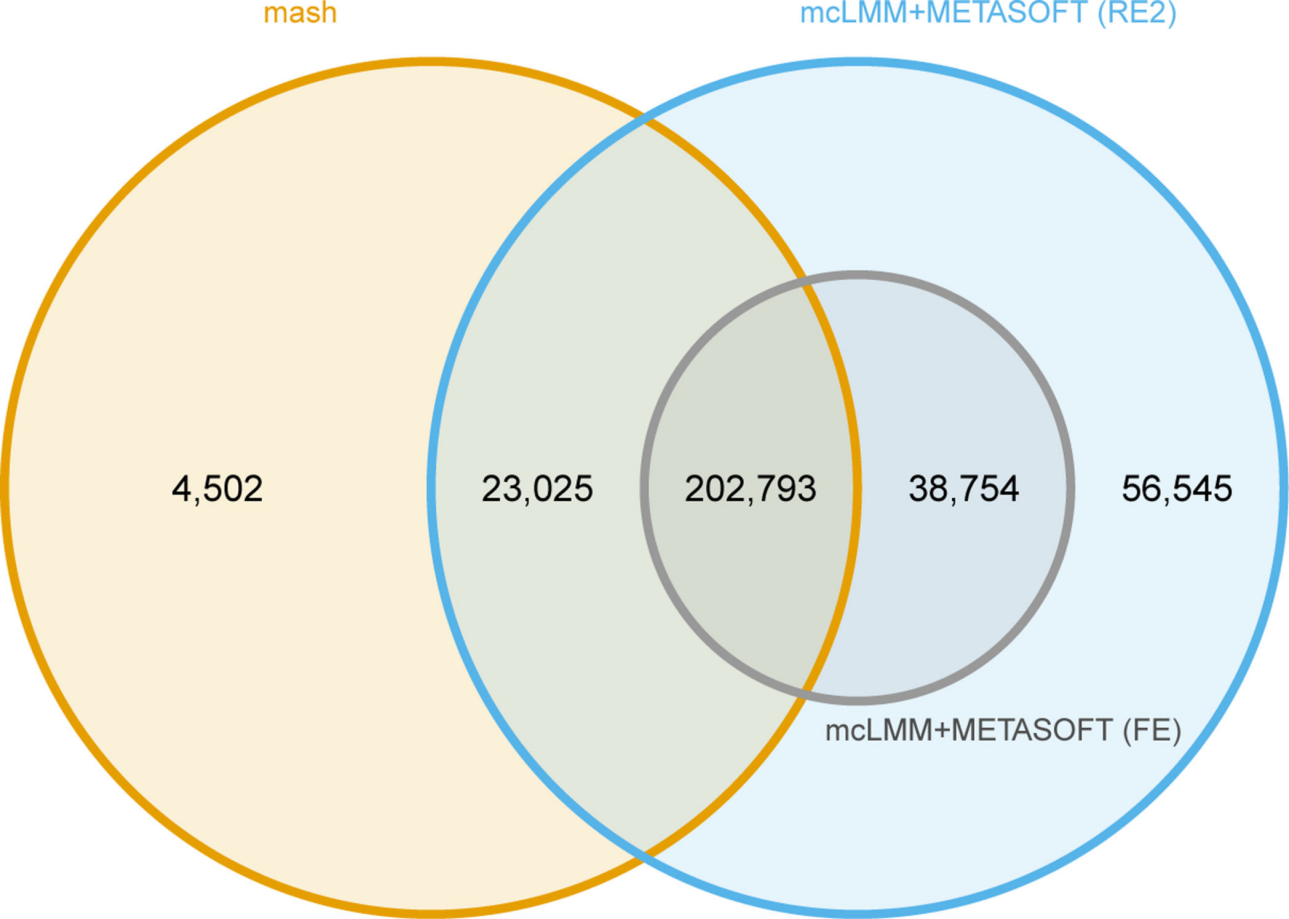
Venn diagram of significant eQTLs identified by meta-analysis methods in the GTEx dataset. We compared mcLMM using the fixed effects (FE) and random effects (RE2) models in METASOFT to **mash**. Note that areas are not proportional to the number of eQTLs in each region. mcLMM+METASOFT (RE2) identified a total of 321,117 significant associations that contained 225,818 eQTLs identified by **mash**.

Next, we compared the number of significant associations identified in the GTEx dataset. The mash approach utilized gene-SNP effect sizes estimated by the GTEx consortium within each tissue independently. Concordant with our simulations, we observed that the Meta-Tissue approach, utilizing mcLMM for vast speedup, identified more significant eQTLs than **mash** (Figure 3). These associations allow researchers to better understand the link between genetic variation and complex phenotypes through possible mediation of gene expression.

### 3.3 mcLMM scales to millions of samples across related phenotypes

As a practical application of the efficiency of mcLMM, we performed a multiple phenotype GWAS in the UK Biobank. A multiple phenotype GWAS associates SNPs with several related phenotypes in order to increase the effective sample size for greater power, under the assumption that the phenotypes are significantly correlated. For our analysis, we combined HDL and LDL cholesterol, Apolipoprotein A and B, and triglyceride levels across 323,266 unrelated caucasian individuals in the UK Biobank. In total, 1,616,330 observations of these related phenotypes were fit as responses in the LMM.

**Figure 4:**
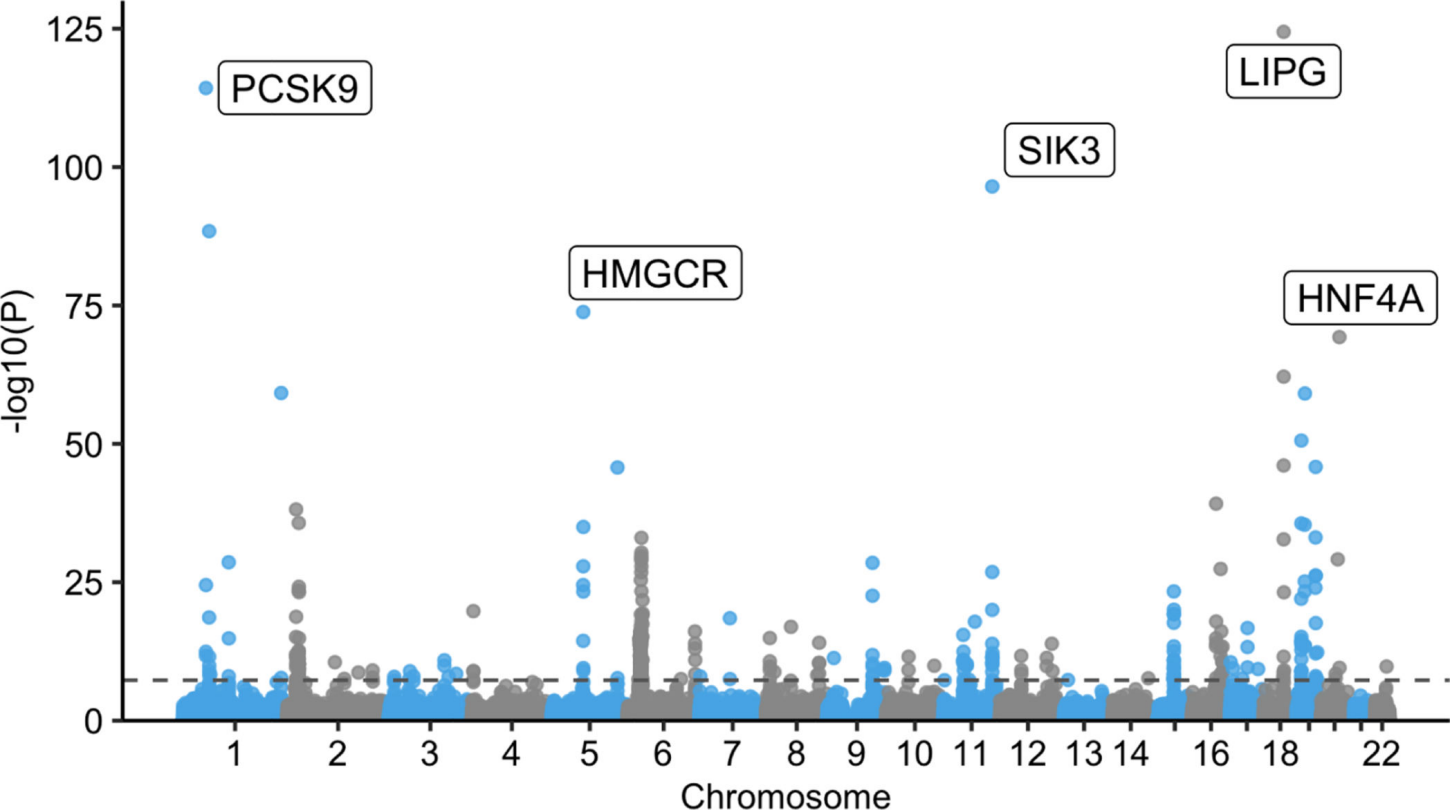
Multiple phenotype GWAS results from UK Biobank. Five phenotypes (LDL cholesterol, HDL cholesterol, Apolipoprotein A, Apolipoprotein B, and triglyceride levels) were used as responses in the mcLMM framework. The model was fit with 1,616,330 observations from 323,266 unrelated Caucasian individuals. In total, 211,642 SNPs were tested with an additional 14 covariates. Each test required around 2 seconds to run on a 32GB machine and was parallelized over each chromosome. The -log10 of the p-values are plot on the y-axis and genomic positions on the x-axis. The horizontal dashed line indicates the genome wide significance level at *p =* 0.05/1e6. The top hit for 5 different chromosomes is annotated with the gene containing the SNP. These genes have been previously identified as associated with a subset of these phenotypes.

The mcLMM approach completed this analysis over 211,642 SNPs with an additional 14 covariates, parallelized over each chromosome, within a day. Each chromosome was analyzed on a single core machine with 32 GB of memory, with each test taking around 2 seconds to complete. We identified several significant loci, a subset of which replicate previous findings for specific phenotypes included in the model, such as HDL cholesterol [25] (Figure 4). Existing approaches, namely EMMA and GEMMA, require orders of magnitude more memory to begin this analyses and could not be run on the available computational resources.

## 4 Discussion

We presented mcLMM, an efficient method for fitting LMMs used for multiple-context association studies. Our method provides exact results and scales linearly in time and memory with respect to sample size, while existing methods are cubic. This efficiency allows mcLMM to process hundreds of thousands of samples over several contexts within a day on minimal computational resources, as we showed in simulation and in the UK Biobank. The association parameters learned by mcLMM can further be utilized with the METASOFT framework to provide powerful meta-analysis of the associations, as we showed in the GTEx dataset.

Previous approaches have derived related speedups for LMMs when the matrix *K* is low rank, such as in the case when multiple samples are genetically identical or clustered in genome wide association studies as described in FaST-LMM [10]. In this approach, the authors show that the likelihood function can be evaluated in linear time with respect to the number of individuals after singular value decomposition of a matrix that is also linear with respect to the number of individuals. Other work has similarly used block structures and Kronecker refactorizations in studies with structured designs, such as multi-trait GWAS, to significantly speed up these approaches as well [9, 13].

Our approach builds upon these findings and we optimize the method specifically for the low rank matrix with known eigenvalues described in the model, thus avoiding any spectral or singular value decompositions. Furthermore, when there is no missing data, our method can compute the optimal model parameters with a closed form solution requiring no iterative optimization of likelihood functions. We also note that mcLMM models covariance across contexts within an individual while the FaST-LMM approach, described above, models covariance across individuals within each context. This specific model fit by mcLMM arises in multiple-context association studies, such as the approach employed by Meta Tissue [20] for identifying eQTLs across tissues utilizing the cubic EMMA algorithm. Applied within this framework for eQTL and multi-trait genome wide association studies, our method provides exact results and scales to hundreds of thousands of samples with minimal computational resources.
